# The Role of Histone Modifications in Plant Priming and Their Analysis by Chromatin Immunoprecipitation

**DOI:** 10.1111/ppl.70915

**Published:** 2026-04-30

**Authors:** Aslihan Temel, Nihal Gören‐Sağlam

**Affiliations:** ^1^ Department of Molecular Biology and Genetics, Faculty of Science Istanbul University Istanbul Türkiye; ^2^ Department of Biology, Faculty of Science Istanbul University Istanbul Türkiye

**Keywords:** chromatin immunoprecipitation, epigenetic modifications, histone modifications, plant priming, plant stress memory, plant stress response, plant stress tolerance

## Abstract

Plants are frequently exposed to adverse conditions. Priming, also known as acclimation or hardening, induces stress memory and prepares plants for future challenges by activating defense and protective mechanisms. For this reason, priming is an effective means to maintain plant yield in the face of climate change. Memory behind the priming is mainly based on epigenetic modifications, for example, histone posttranslational modifications (PTMs) on the priming‐related genes. While histone PTMs are the most diverse group of epigenetic modifications and regulate gene expression via addition of chemical groups to histone amino acids, their characterization is challenging. Chromatin immunoprecipitation (ChIP) is an essential method for the characterization of histone PTMs; however, subject to many challenges, especially in plant samples. This review discusses the current understanding of histone modifications in plant stress and priming and the ChIP methodology and troubleshooting. Yield losses resulting from climate change necessitate the use of priming as an agricultural practice. In order to apply priming, an in‐depth analysis of stress‐ or priming‐induced histone PTMs is essential. ChIP has been extensively used in plant stress studies and has undergone numerous improvements. Although there are more sophisticated methods, ChIP is still regarded as a standard method for the characterization of chromatin profiles. This review aims to support researchers in the utilization of ChIP, particularly, for plant stress and/or priming studies.

## 1 Introduction

Plants are subjected to diverse forms of environmental stresses, the impacts of which are estimated to be exacerbated by climate change (Patel et al. 2024). Due to their sessile nature and often long lifespans, plants are prone to stressful conditions, some of which affect them repeatedly during their lifespan (Mozgova et al. 2019; Chaudhry and Sidhu 2022). Elevated temperatures, particularly, accompanied by drought, pose a great risk to crop yields and eventually food security, and their intensity/duration have been predicted to increase in the future (Mozgova et al. 2019; Janni et al. 2024; Patel et al. 2024). Climate change and increasing human populations necessitate revisiting our agricultural production. The reduction in crop production can be overcome by the utilization of fertilizers and biostimulants, as well as the development of stress‐tolerant crops (Zhang et al. 2018; Janni et al. 2024). Abiotic stresses (e.g., nonoptimal temperatures, drought, flood, salinity) share common consequences including osmotic imbalance, oxidative damage, and altered gene expression (Kumari et al. 2022).

Plants respond to abiotic stress by adjusting water uptake, synthesizing metabolites, regulating antioxidant systems and maintaining the photosynthetic apparatus (He et al. 2018). Central to these processes is the regulation of gene expression and chromatin through an array of epigenetic mechanisms, including histone modifications, histone variants, DNA methylation, ATP‐dependent chromatin remodeling and noncoding RNA (Sharma et al. 2023; Aswathi et al. 2025). As sessile organisms, plants encounter recurrent unfavorable conditions; consequently, they develop “memory” or “stress memory,” a phenomenon where an initial stimulus triggers changes that persist even after the stressor is removed (Lämke and Bäurle 2017; Sharma et al. 2023). Upon the occurrence of stress, same or different, the memory is activated to prompt a faster and enhanced response (Mozgova et al. 2019).

**FIGURE 1:**
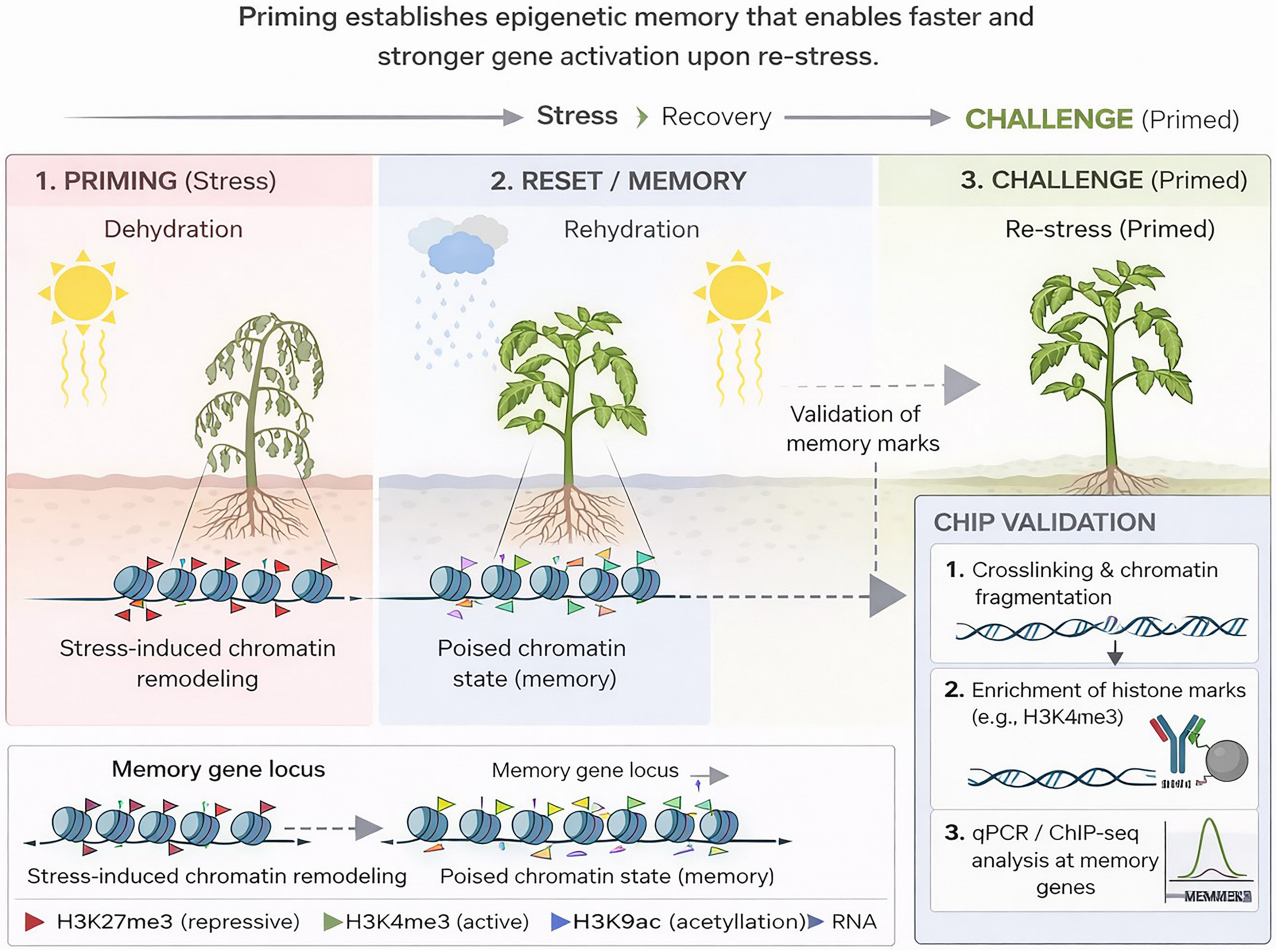
A schematic view of stress memory in plants and analysis of histone PTMs using ChIP. Generated using BioRender, Google Gemini, and ChatGPT.

Stress memory can be categorized as intergenerational, affecting the immediate offspring, or transgenerational, persisting across multiple generations (Oberkofler et al. 2021; Liu, Able, et al. 2022; Bhatt et al. 2026); yet, it may be lost upon exposure to stress‐free conditions, even for one generation (Wibowo et al. 2016). Memory is formed by the interplay of metabolites, signaling molecules, hormones and epigenetic modifiers (Louis et al. 2023; Rossatto et al. 2023; Bhatt et al. 2026). According to Fleta‐Soriano and Munné‐Bosch (2016), drought memory is the combination of epigenomics, transcriptomics, proteomics and metabolomics. Primed plants exhibit transcriptional differences from non‐primed plants as memory genes in primed plants maintain their induced status until a re‐exposure to stress. However, most of the stress memory relies on the epigenetic machinery (Mozgova et al. 2019; Liu, Able, et al. 2022; Aswathi et al. 2025) as the maintenance of the primed states is achieved by chromatin modifications that alter chromatin structure, which enables the transcriptional machinery to access the genes to be induced (Aswathi et al. 2025). When faced with stress, plants are primed to store stress‐induced changes as memory, enabling them to respond in a faster, stronger, and/or sensitized fashion upon exposure to a recurrent stress (Figure 1) (Lämke and Bäurle 2017; Turgut‐Kara et al. 2020; Harris et al. 2023; Aswathi et al. 2025).

**FIGURE 2:**
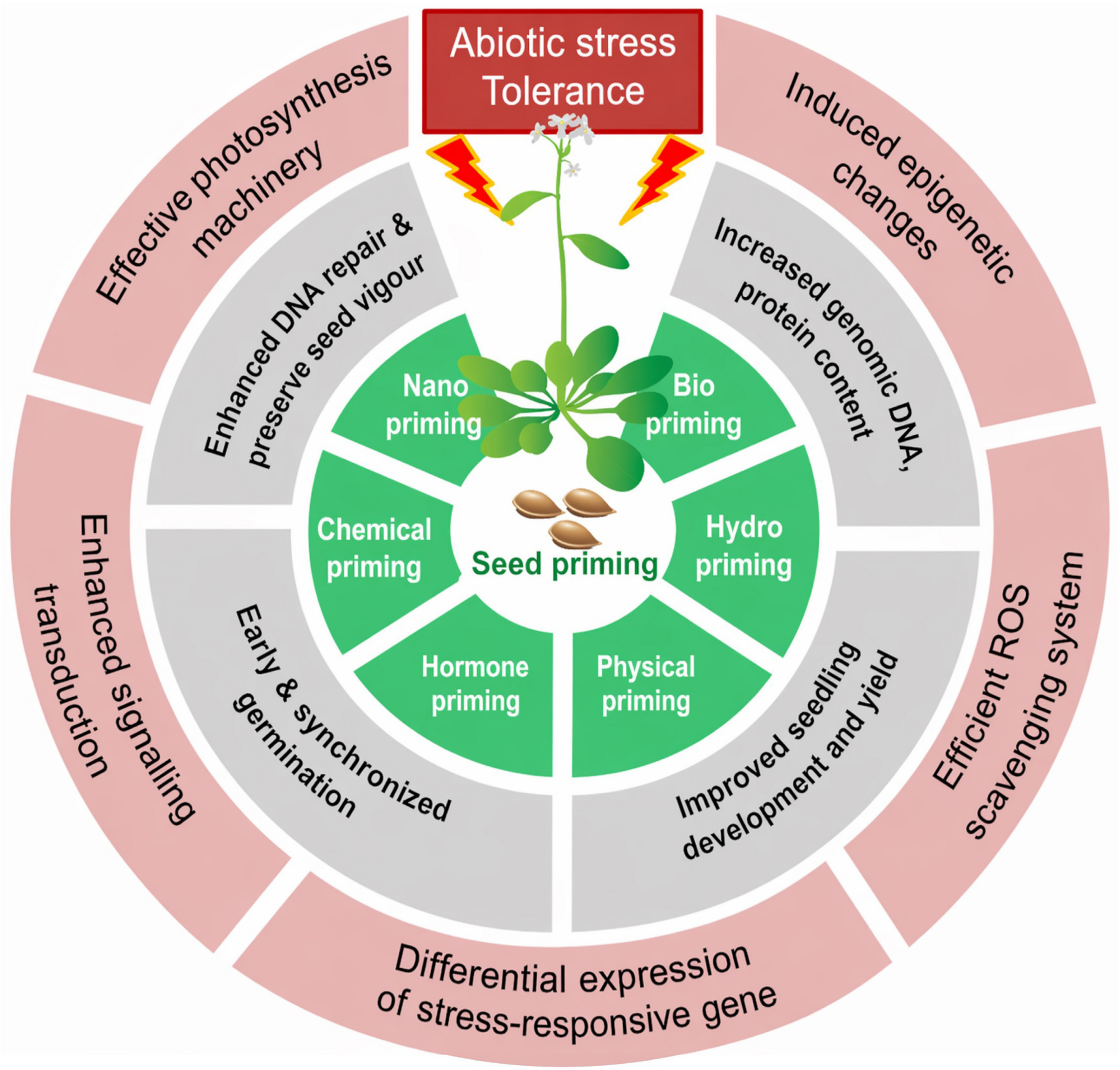
An overview of the effects of seed priming with various priming agents. An overview of the effects of seed priming with various priming agents. Priming can be achieved by several approaches such as bio, chemical, hormone, hydro, nano, physical and stress. Priming affects germination, DNA repair, increased DNA and protein content and improves through increased stress tolerance modulated by epigenetic changes, differential gene expression, signaling, better scavenging of reactive oxygen species (ROS) and improved photosynthesis.

Stress memory holds significant potential for breeding purposes. Priming, often referred to as acclimation or hardening within the context of abiotic stress, facilitates crop improvement through the induction of a stress memory (Conrath et al. 2006; Harris et al. 2023). Stress memory provides tolerance to an additional stress in the current or even subsequent generations (Rossatto et al. 2023; Bhatt et al. 2026). Furthermore, primed plants can exhibit cross‐stress tolerance, where an initial stimulus provides protection against a distinct subsequent stressor (Rossatto et al. 2023). In addition to stress, plants can be primed by chemical compounds, for example, hydrogen peroxide or melatonin (Savvides et al. 2016; Yaycili et al. 2025). Whether induced by stress or chemicals, priming offers a promising direction for sustainable agriculture under climate stress (Aswathi et al. 2025). Figure 2 summarizes the overview of the effects of seed priming with various priming agents on plant growth, development, and stress tolerance via activating specific physiological and molecular mechanisms (Louis et al. 2023).

To study the chromatin modifications that alter chromatin structure during priming/stress memory, chromatin immunoprecipitation (ChIP), which maps the genomic location of specific histone proteins and modifications (Huang et al. 2015), is the cornerstone of chromatin research (Sidoli et al. 2012). While histones are abundant in the cell and relatively easy to isolate, their analysis remains a challenging task due to several reasons (Shechter et al. 2007; Holt et al. 2021). In this review, we briefly describe plant stress memory and priming before focusing on chromatin modifications in these processes. Then, we specifically summarize the two most prominent modifications, acetylation and methylation, and provide an in‐depth discussion of ChIP methodology, its limitations in plant systems, and technical optimizations. Finally, we outline how an improved understanding of stress‐related histone modifications can help breeding programs and propose technical suggestions to improve ChIP and its successor methods.

## 2 Chromatin Modifications

Chromatin is the packaged form of DNA with proteins, composed primarily of histones, and RNA (Bernstein and Allis 2005; Noberini et al. 2022). The fundamental unit of chromatin is the nucleosome, which consists of 147 bp of DNA wrapped around two copies of four canonical histones (H2A, H2B, H3, and H4). Another histone, H1, stabilizes the nucleosome by linking it to the DNA strand (Sidoli et al. 2012). The density of nucleosomes and/or any histone is related to the transcription status. For instance, upstream regions of highly expressed genes exhibit low H3 occupancy (Oh et al. 2008; Van Dijk et al. 2010). Histones undergo several posttranslational modifications (PTMs) such as acetylation, ADP‐ribosylation, crotonylation, deamination, methylation, phosphorylation, SUMOylation, and ubiquitination (Strahl and Allis 2000). Beyond PTMs, histones also exist as variants; one of the best‐characterized is the centromeric H3, also known as CENP‐A (Le et al. 2025). The specific location, type, and combination of these PTMs collectively constitute the histone code (Strahl and Allis 2000). Histone PTMs can block or facilitate the formation of other PTMs (Le et al. 2025). The influence of PTMs on histone structure might be greater than that of a mutation due to the abundance of PTMs in histones (Huang et al. 2015). Consequently, the histone code determines the accessibility state of a DNA region in terms of function (e.g., transcription, DNA repair, etc.). PTMs are established and removed by enzymes known as writers and erasers, respectively, and are recognized by reader proteins (Noberini et al. 2022; Le et al. 2025). Reader proteins mediate the outcome by recruiting other proteins involved in multiple DNA‐associated processes, including transcription, heterochromatinization, DNA repair, and replication.

In plants, there are 3 DNA methyltransferase activities that catalyze the methylation of a cytosine residue in three distinct sequence contexts, CG, CHG, and CHH. Unlike in plants, DNA methylation in mammals occurs primarily in the CG context (He et al. 2011). Nevertheless, in both systems, DNA methylation is more abundant in repetitive elements (e.g., transposable elements; TEs) and also occurs in the promoter regions of genes and is associated with the silencing of genes and intergenic regions (Vanyushin and Ashapkin 2011).

Phenotypic changes that are stable through cell division and independent of DNA sequence changes are defined as epigenetics (Lloyd and Lister 2022). These changes, including DNA methylation, histone PTMs, and histone variants, are dynamic and essential for all aspects of plant life, particularly, stress response, enabling plants to cope with harsh conditions and holding a significant role in crop improvement (Liu et al. 2015; Samantara et al. 2021). In addition to stress response, several developmental processes are regulated by genetic and epigenetic mechanisms and environmental cues (Hemenway and Gehring 2023). One of the loci demonstrated to be epigenetically regulated depending on environmental conditions is Flowering Locus C (*FLC*). Cold exposure triggers transcription of the FLC antisense transcript by causing the loss of H3K36me3 and accumulation of H3K27me3 (Bastow et al. 2004; Fang et al. 2020).

Stress conditions alter chromatin structure in plants at all levels, usually resulting in decondensation of heterochromatin and induction of stress‐response genes (Mozgova et al. 2019). Conversely, stress‐responsive gene expression may lead to changes in chromatin structure (Secco et al. 2015). Certain metabolites (e.g., nicotinamide adenine dinucleotide [NAD], *S*‐adenosylmethionine [SAM], and acetyl coenzyme A) are directly connected to the epigenetic machinery, particularly, through the enzymes involved in histone modifications. For this reason, altered levels of these metabolites are likely to trigger epigenetic modifications (Vriet et al. 2015). Most of the work supporting the connection between metabolism and epigenetics has been obtained in animals (Lu and Thompson 2012). Yet, plant‐based studies have provided comparable insights. For instance, 1C metabolism is essential for DNA and histone methylation. Similarly, intermediates of the tricarboxylic acid (TCA) cycle regulate the enzymes involved in demethylation reactions. Furthermore, cellular redox status, TCA, and fatty acid β‐oxidation affect histone acetylation and deacetylation reactions. The capacity of plants to rapidly adapt to fluctuating environments might be facilitated by the regulation of their diverse metabolites, most of which are secondary metabolites (Song et al. 2026). Identification of metabolite(s) involved in particular epigenetic changes might help priming studies. In their review paper, Lu et al. (2023) exhaustively discussed the interplay between plant metabolism and the epigenome.

Stress‐induced transcription factors can directly recruit and activate histone‐modifying complexes (e.g., H3K4 methyltransferase; Song et al. 2015) or carry out the eviction of particular variants of histones from downstream genes that are supposed to be activated (Cortijo et al. 2017). An example is the signaling of abscisic acid (ABA), level of which increases under harsh conditions. Under nonstress conditions, the chromatin remodeling complex BRM maintains the silencing of *ABI5* gene, which is at the core of ABA signaling, by keeping a nucleosome at its transcription start site (Han et al. 2012). BRM, in turn, is inhibited by phosphorylation induced upon ABA perception, allowing for the activation of the ABA response (Peirats‐Llobet et al. 2016). Stress conditions also induce DNA methylation or demethylation events that might be genotype‐ and tissue‐specific and remain after the stress (Wang et al. 2011). Some stress‐responsive genes are usually kept silent by DNA methylation under optimal conditions in order to prevent their expression from interfering with growth and development (Vriet et al. 2015).

Late differentiation of plant germline from somatic tissues, which may have been exposed to stress and therefore acquired epigenetic marks, explains the transmission of long‐term effects of epigenetic signatures. In addition, plants exhibit vegetative reproduction, which further amplifies the potential for inheritance (Mozgova et al. 2019). When taking the nature of plants into consideration, the association between stress response, stress memory and epigenetic changes creates a multifaceted landscape and necessitates deeper analyses. Although DNA is a key player in epigenetics, there are several reasons to highlight the versatility and complexity of histones. For instance, there is a vast array of histone PTMs compared to DNA methylation. In addition, histone PTMs correlate directly with long‐term cellular outcomes and specific histone marks (e.g., lysine methylation) exhibit high stability. Furthermore, DNA methylation is often dependent on histone PTMs (Sidoli et al. 2012; Huang et al. 2015), while certain histone PTMs conversely rely on the DNA methylation machinery (Fuks et al. 2003). Therefore, understanding histone dynamics is essential for chromatin studies, as well as plant stress memory.

## 3 Status of Histone Modifications During Stress and Priming

Histone modifications serve as molecular scaffolds, recruiting transcription factors and readers (Chhatwal et al. 2024; Kim 2025). Under stress conditions, plants undergo epigenetic alterations, particularly histone acetylation, decreased DNA methylation and loss of heterochromatin on a global scale, alongside locus‐specific changes in DNA and histone methylation (López et al. 2022; Huang et al. 2023; Xie et al. 2019; Yung et al. 2024; Zi et al. 2024; Zhuang et al. 2026). These chromatin changes alter the accessibility of the memory genes to the transcriptional machinery (Nair et al. 2022). Beyond acetylation and methylation, ubiquitination, phosphorylation and several other PTMs also influence diverse plant processes, including stress response and tolerance (Nunez‐Vazquez et al. 2022; Chhatwal et al. 2024). Phosphorylation of particular amino acids increases the negative charge of the histones and destabilizes the nucleosome structure. Histone monoubiquitination is the addition of ubiquitin, a 76‐amino acid polypeptide, to lysines and increases the stability of the protein by inhibiting its polyubiquitination, which typically results in proteasomal degradation of the protein. Histone monoubiquitination might be associated with transcriptional activation or repression depending on the location of monoubiquitinated lysine (Chen et al. 2022).

### 3.1 Histone Acetylation

As positively charged amino acids, lysines provide histones with a high electrostatic affinity for DNA. The acetylation of lysine residues (K) by histone acetyltransferases (HATs) weakens the histone‐DNA interactions by neutralizing their positive charge and, therefore, has a major impact on chromatin structure, driving a transition from a closed to an open chromatin state (Le et al. 2025). A hallmark example is H4K14 acetylation, which significantly inhibits chromatin compaction (Robinson et al. 2008). H3K9ac, along with H3K27me1, indicates chromatin compartments in tomato (Huang et al. 2023). A less compact chromatin structure facilitates the accessibility of DNA to proteins and protein complexes, such as those involved in transcription, thereby enhancing transcription (Narita et al. 2018; Le et al. 2025). Histone acetylation is reversed by histone deacetylases (HDACs) (Le et al. 2025), which comprise 3 families in plants, including the HD2 family, a plant‐specific HDAC, in addition to the RPD3 and Sir2 families (Tahir and Tian 2021). Acetylation of H3K9 maintains an open transcriptionally active state by blocking repressive methylation, while its deacetylation is essential for its subsequent methylation (Rice and Allis 2001). Bromodomain units bind to acetylated lysines and are present in chromatin remodelers, ATP helicases, histone methyltransferases (HMTs), and HATs (Marmorstein and Zhou 2014). The activity of both HATs and HDACs is tethered to the metabolic state of the cell. HATs utilize acetyl coenzyme A as the acetyl group donor, whereas deacetylation of histones by HDACs produces acetate. Acetyl coenzyme A is a key metabolic intermediate and is derived from several processes such as fatty acid β‐oxidation (Lu et al. 2023). SIR2, a type of HDAC, utilizes NAD^+^ and produces nicotinamide, an inhibitor of poly‐(ADP‐ribose) polymerase (PARP) enzymes. PARPs use NAD^+^ to transfer ADP‐ribose chains to proteins and are involved in the maintenance of DNA integrity and cell death in case DNA is damaged beyond repair (Salech et al. 2020; Navas and Carnero 2022; Lu et al. 2023). Therefore, histone acetylation interconnects genome stability and metabolism.

**FIGURE 3:**
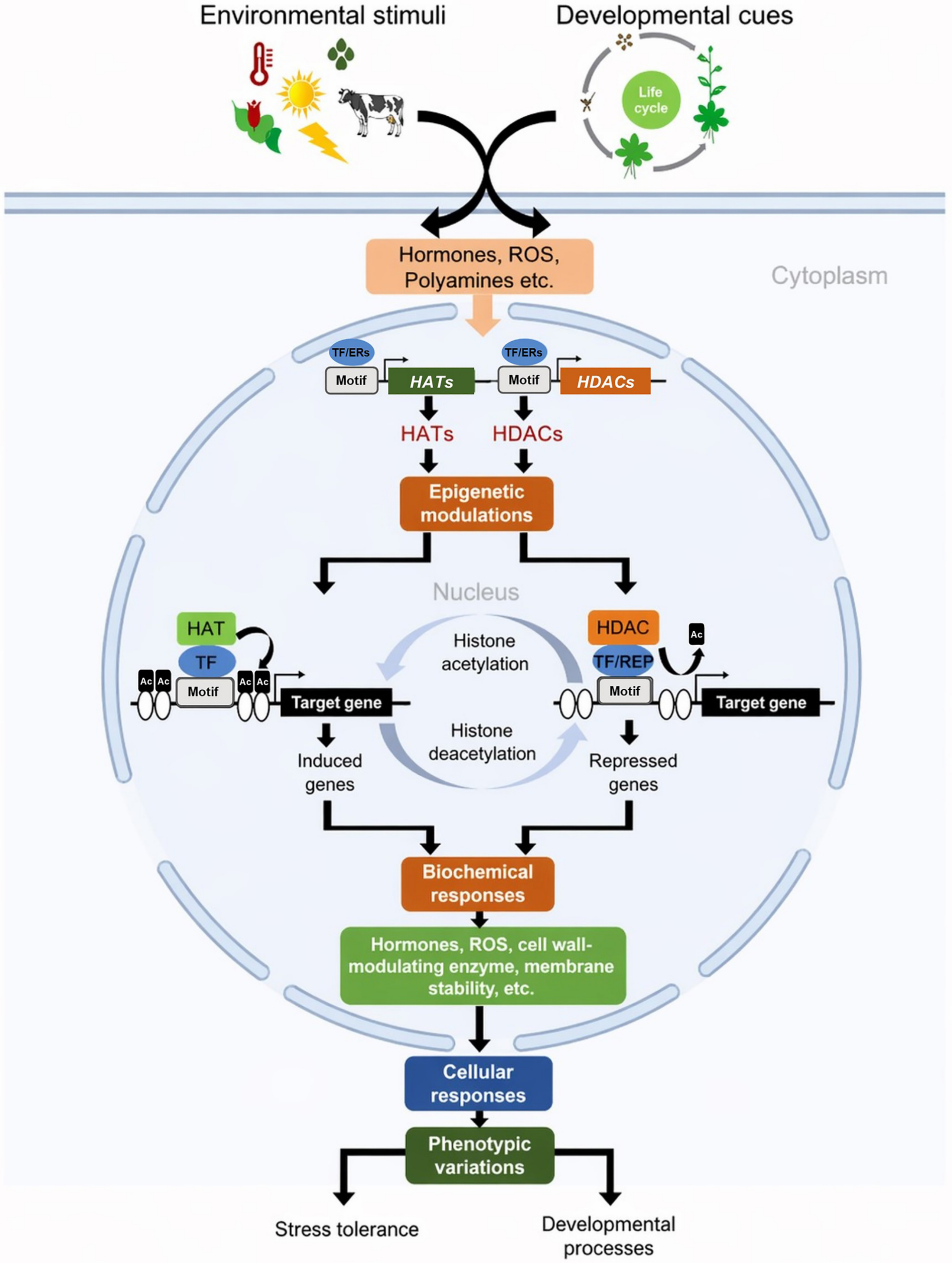
An overview of histone acetylation‐mediated epigenetic regulation of developmental processes and stress tolerance in plants. The developmental and environmental stimuli trigger signaling cascade that leads to the expression of HAT and HDAC genes (Kumar et al. 2021). Ac: acetyl; ER: epigenetic regulator; HAT: histone acetyltransferase; HDAC: histone deacetylase; ROS: reactive oxygen species; RP: repressor protein; TF: transcription factor.

Histone acetylation is a transient and highly dynamic process. Histone acetylation marks can be deposited on stress‐responsive genes 4 h after stress onset and removed within 24 h of stress recovery. The induction of histone acetylation correlates with the expression of the stress‐responsive genes, while histone deacetylation is associated with gene silencing. Loss of nucleosome occupancy, however, might require a longer stress duration and appears to occur independently of histone acetylation status (Pavangadkar et al. 2010). The involvement of HATs and HDACs in stress response and priming is context‐dependent, with their functions dictated by the stress type and/or duration. For instance, GCN5, a HAT, is essential for thermotolerance (Hu et al. 2015) but not for cold tolerance (Pavangadkar et al. 2010). Conversely, HDACs are usually viewed as negative regulators of stress tolerance (Zheng et al. 2016) and most of them are repressed under osmotic stress (Hu et al. 2009). A notable example is HD2C. It plays an essential function in the repression of heat‐induced genes to regulate heat stress response in *Arabidopsis* (Buszewicz et al. 2016). Yet, several HDACs were shown to be induced by drought but not by salt stress (Zhao et al. 2015). Transposon‐derived SANT proteins regulate gene expression by acting as HDACs and regulate heat tolerance (Zhou et al. 2024). The interconnection between stress tolerance, priming and metabolism in plants was demonstrated by Kim et al. (2017). HDA6 is an essential component of a stress tolerance pathway linked to the acetate biosynthetic pathway. This finding identified acetic acid as a priming agent (Kim et al. 2017). An HDAC inhibitor (trichostatin A; TSA) and the arginine‐metabolism intermediate *N*‐acetylglutamic acid have both been utilized as chemical priming agents, increasing H3K9ac levels (Yung et al. 2022; Hirakawa et al. 2023). Salinity priming also caused alterations in H3K9ac levels (Yung et al. 2022). Figure 3 shows the histone acetylation‐mediated epigenetic regulation of developmental processes and stress tolerance in plants (Kumar et al. 2021).

### 3.2 Histone Methylation

Unlike histone acetylation, which alters chromatin structure through charge neutralization, histone methylation is biophysically neutral as the methyl group is small and unlikely to affect the charge of amino acids. Nevertheless, it remodels the chromatin structure and gene expression by creating binding sites for the regulatory proteins that recognize the methylated residues (Strahl and Allis 2000). HMTs transfer methyl groups from the methyl group donor SAM to lysine and arginine residues. Arginines can carry one or two methyl groups, and lysines can have up to three methylations (Strahl and Allis 2000; Nunez‐Vazquez et al. 2022). Several protein domains (e.g., chromodomain) can bind to certain methylated lysines (Martin and Zhang 2005). The removal of methyl groups from lysine and arginine is catalyzed by histone demethylases (HDMs), including lysine‐specific histone demethylase 1 (LSD1) and Jumonji C (JmjC) domain‐containing enzymes (Strahl and Allis 2000; Nunez‐Vazquez et al. 2022). Similar to HATs and HDACs, HDMs also act as metabolic sensors. LSD1 and JmjC HDMs utilize flavin adenine dinucleotide and α‐ketoglutarate, respectively. In addition, TCA cycle intermediates, like succinate and fumarate, are inhibitors of α‐ketoglutarate‐dependent HDMs. Consequently, histone methylation status is sensitive to TCA and one‐carbon metabolism (Su et al. 2016; Lu et al. 2023).

The best‐characterized histone methylation events are the trimethylation of H3K4 (H3K4me3) and H3K27 (H3K27me3) residues (Nunez‐Vazquez et al. 2022). While these marks often indicate distinct genomic regions, they can co‐exist in certain genomic regions known as bivalent domains. H3K27me3, accompanied by a moderate amount of other activation marks, is found in coding gene regions (Sequeira‐Mendes et al. 2014). H3K4me3 is predominantly enriched in upstream regions of active genes, where it facilitates transcription by recruiting transcription initiation factors to promoters and preventing *de novo* DNA methylation (Ooi et al. 2007; Xiao et al. 2016; Millán‐Zambrano et al. 2022; Nunez‐Vazquez et al. 2022). SET‐domain‐containing enzymes (e.g., arabidopsis trithorax‐like factor ATX1) are the major H3K4 trimethyltransferases in plants (Le et al. 2025). Although the enzymes that catalyze H3K4 trimethylation are associated with transcriptional machinery and H3K4me3 and H3K9ac are often positively correlated, H3K4 methylation might not be essential for transcription; instead, it might be a result of transcription (Martin and Zhang 2005; Henikoff and Shilatifard 2011; Yu et al. 2025).

H3K4me2/3 accumulates at the memory genes associated with heat and drought stresses (Ding et al. 2012; Kim et al. 2012; Lämke et al. 2016). H3K4me3 is the most affected histone methylation mark in *Arabidopsis* plants under water‐deficient conditions (Van Dijk et al. 2010). Under cold stress, genes that gain H3K4me3 exhibit higher levels of induction, although H3K4me3 is not essential for the initiation of transcription (Faivre et al. 2024). ATX1 is essential for both ABA‐dependent and ‐independent dehydration stress tolerance (Ding et al. 2011). Conversely, other SET‐domain enzymes limit ABA signaling under dehydration stress by adding H3K4me3 on the negative regulator of ABA signaling (Liu et al. 2018). JMJ17, a HDM that acts on H3K4me1/2/3, is a critical regulator of dehydration response, as its deficiency causes ABA hypersensitivity (Huang et al. 2019). H3K4me3 is an indicator of the memory genes involved in dehydration stress (Liu et al. 2014) (Table 1).

**TABLE 1:** Regulation of histone PTMs in plants under stress or chemical priming and the histone PTM‐related outcomes.

Histone mark	Stress	Plant species	Molecular and/or physiological outcomes	Reference, additional remarks
H3K4me1, H3K4me2, H3K4me3	Drought stress, withholding irrigation until a RWC of 65% was reached	*Arabidopsis thaliana*	Down‐regulated genes are marked with H3K4me1; H3K4me3 is correlated with transcription status; H3K4me2 has minor effects	Van Dijk et al. 2010, upstream of highly‐expressed genes contain less H3
H3K4me3	Osmotic or dehydration stress, 200 mM mannitol or 30% PEG for 12 days; soil dehydration by withholding irrigation for 14 days, rewatering for 3 days	WT and *atx1* mutant of *A. thailana*	ATX1, a H3K4 trimethyltransferase, is essential for dehydration tolerance by regulating NCED3 expression and slowing transpiration	Ding et al. 2011, ABA‐independent genes are also controlled by ATX1
H3K4me3	Dehydration stress, removing plants from soil, and then air‐drying for 2 h, recovery (R1) by placing plants in humid chambers for 22 h with their roots in a few drops of water. Stress and recovery steps were repeated for an additional 2 rounds	WT and *atx1* mutant of *A. thaliana*	After stress, some genes (*RD29B* and *RAB18*) have basal transcription but maintain H3K4me3 and (Ser5P) Pol II	Ding et al. 2012, the first report of stalled Pol II (phosphorylated) at plant genes
H3K4me3, H3K9ac	Dehydration stress, keeping plants on dry plastic dishes for 4 h; rehydration by adding water for 1, 2, 3, or 5 h	*A. thaliana*	H3K4me3, RNA pol II and H3K9ac are enriched on dehydration‐induced genes but RNA pol II and H3K9ac are removed rapidly after recovery	Kim et al. 2012, nucleosome density (H3 occupancy) differentiate drought‐inducible genes (*RD29A*) from the rehydration‐inducible gene (*ProDH*)
H3K4me2, H3K4me3	Heat stress (37°C for 60 min, 23°C for 90 min, and 44°C for 45 min); recovery for 4, 28 or 52 h	*A. thaliana*	H3K4me3 and H3K4me2 accumulate at *HSP* loci; H3K4me2 is added at a later stage	Lämke et al. 2016, sustainment of H3K4me3 and H3K4me2 depends on an HSF TF
H3K4me3, H3K27me3	Cold stress, at 4°C for 3 h or 3 days	WT and *clf‐28* mutant of *A. thaliana*	Genes rather gain H3K4/27me3 instead of losing; H3K4me3 is more prominent than H3K27me3, which is more stable than H3K4me3, H3K4me3 and H3K27me3 are associated with stress‐responsive and developmental genes, respectively	Faivre et al. (2024), histone methylation is slightly correlated with gene expression; H3K27me3 does not affect either H3K4me3 or cold tolerance
H3K4me3	Drought stress, withholding irrigation for 14 days, re‐watering for 2 days	*WT* and *ATX* mutants of *A. thaliana*	ATX4 and ATX5 di‐methylate and trimethylate H3K4 and regulate ABA responses by regulation of the H3K4me3 status at *AHG3*, an essential negative regulator of ABA signaling	Liu et al. (2018), ATX4 and ATX5 are essential for RNA Pol II occupancy and limit ABA response
H3K4me3	Drought stress, withholding irrigation for 14 days, rewatering for 3 days	*WT* and *jmj17* mutants of *A. thaliana*	JMJ17 removes H3K4me3 from *OST1*, a Ser/Thr protein kinase involved in dehydration and ABA responses	Huang et al. (2019), Dehydration stress and ABA down‐regulate the transcription of JMJ17, which represses dehydration stress response
H3K4me3, H3K27me3	Dehydration stress, removing plants from soil, and then air‐drying for 90 min, recovery (R1) by placing plants in humid chambers for 22 h with their roots in a few drops of water. Stress and recovery steps were repeated for additional 2 rounds	WT and *clf* mutants of *A. thaliana*	H3K27me3 does not prevent transcription, H3K4me3 and H3K27me3 are independent	Liu et al. (2014), CLF decreases transcription rate
H3K27me3	12‐day‐old plants grown on media supplemented with 10 mM NH_4_NO_3_	WT and *HNI9, wtNL* mutants of *A. thaliana*	HNI9/AtIWS1 is a component of RNA Pol II complex and responsible for H3K27me3 deposition on *NRT2.1*, the root nitrate transporter	Widiez et al. (2011), N supply represses *NRT2.1* transcription by depositing H3K27me3 on it
H3K27me3	UV stress, 2 Wm^−2^ of UV‐B (311 nm) for 1 h until flowering	WT and several mutants of *A. thaliana*	UV stress decreases H3K27me3 on flowering time genes (*miR156*, *FLC*)	Dotto et al. (2018), UVR8 is essential for UV‐B‐induced delay in flowering time
H3K4me3, H3K27me3	Two types of heat stress, acclimation (37°C for 20 min) and heat‐shock treatment (43.5°C for 60 min)	WT and several mutants (*jmj*, *hsp* etc.) of *A. thaliana*	H3K27me3 demethylase is necessary for thermotolerance; H3K4me3 deposition and H3K27me3 removal may be interconnected	Yamaguchi et al. (2021), H3K4me3 is not necessary for activation of HSP genes
H3K4me3, H3K9ac, H3K18ac, and H3K27ac	Heat stress, plants were treated at 45°C for 1 or 6 h	(WT) and *hsfa1a* knock‐down transgenic lines of tomato	Heat stress had little effect on the accumulation of these PTMs; distal and proximal REs display different chromatin signatures	Huang et al. (2023), an improved Hi‐C method; heat stress results in chromatin spatial reorganization mediated by HSFA1a
H3K27me3	Shade treatment (R, ~30 μmol m^−2^ s^−1^; B, ~20 μmol m^−2^ s^−1^; FR, ~65 μmol m^−2^ s^−1^) for 1 or 7 h	WT and several mutants of *A. thaliana*	shade‐induced genes lose H3K27me3 mark; and H3K27me3 demethylation is required for shade avoidance memory	Cheng et al. (2024), PIF7 interacts with REF6, a histone H3K27 demethylase
H3K9me2	Drought stress, withholding irrigation until a RSWC of 15% was reached	*WT* and *jmj27* mutants of *A. thaliana*	JMJ27, a H3K9me2 demethylase is necessary for drought stress response by activating genes and inducing stomatal closure	Wang et al. (2021), drought stress inhibits proteasome‐mediated degradation of JMJ27
H3K9ac, H3K14ac	Cold stress, growing plants at 4°C for 0, 4, or 24 h. One group was acclimated at 4°C for 24 h and then de‐acclimated at 22°C for 24 h	WT and several mutants of *A. thaliana*	H3 acetylation increases while H3 occupancy decreases at COR gene promoters in response to cold stress but are reversed upon deacclimation	Pavangadkar et al. (2010), histone acetylation alone is not sufficient for cold‐stress induction of transcription
H3K9ac, H3K14ac	Heat stress, growing plants 40°C for 1 h	Wassilewskija and Columbia lines and *gcn5* and *uvh6* mutants of *A. thaliana*	GCN5 affects the acetylation of H3K9/14 at the promoters of heat stress‐responsive genes	Hu et al. (2015), certain genes that were down‐regulated in gcn5 mutant are not enriched in WT plants
H3K9ac	Salt and drought stresses, drought stress was achieved by PEG‐infused medium whose Ψ_w_ was −1.2 MPa, salt stress was achieved by NaCl‐infused medium whose Ψ_w_ was −1.2 MPa	WT and *hda9‐1* and *hda9‐2* mutants of *A. thaliana*	HDA9 confers sensitivity against salt and drought stresses by reducing H3K9ac accumulation on stress‐responsive genes	Zheng et al. (2016), HDA9‐mediated regulation of several genes, e.g., *HSP90.2* is independent of histone acetylation
H3K14ac, H4K16ac	Heat stress, growing plants in 38°C for 1 h	WT and several mutants of *A. thaliana*	HD2C prevents H4K16Ac on *HSFA3* and *HSP101* and maintains their silence until heat stress	Buszewicz et al. (2016), HD2C‐mediated regulation of several genes, e.g., *APX2* is independent of histone acetylation; BRM, the catalytic subunit of a SWI/SNF complex, acts with HD2C
H4Ac	Drought stress, withholding irrigation for 6 or 12 days	WT and several mutants of *A. thaliana*	Drought decreases binding levels of HDA6 to its target regions, e.g., *ALDH2B7* and H4Ac accumulates	Kim et al. (2017), *hda6* mutants have higher endogenous acetic acid levels
H3K4me2, H3K4me3, H3K9ac, H3K18ac	Salt stress, growing plants in 0.5× Hoagland's solution containing 0.3% (w/v) NaCl for 0.5, 1, 2, 8, or 24 h	*Glycine max* cultivar C08	H3K4me3 is the most prominent mark between primed and nonprimed plants	Yung et al. (2022), Histone PTMs are associated with gene transcription, modulation of H3K9ac levels by TSA treatment
H4ac	NAG treatment, incubating seedlings with 0.4 mM NAG for 2 h	*A. thaliana Col‐0* and rice cv. Nipponbare	NAG increased H4 acetylation and induced the expression of TFs involved in oxidative stress response	Hirakawa et al. (2023), NAG induced the transcription of HATs and enhances oxidative stress tolerance in *A. thaliana* and rice

Abbreviations: B: blue light; FR: far‐red light; Hi‐C: high‐throughput chromosome conformation capture; HSF: heat‐shock factor; HSP: heat‐shock protein; NAG: *N*‐acetylglutamic acid; PEG: poly‐ethylene glycol; PIF7: phytochrome‐interacting factor 7; R: red light; REF6: relative of early flowering 6; RE: regulatory element; RSWC: relative soil water content; RWC: relative water content; TF: transcription factor; TSA: trichostatin A; Wm^−2^: watts per square meter; Ψ_w_: water potential.

H3K27me3 is found in inactive genes and excluded from heterochromatin regions, which are instead marked with H3K9me2. Similar to H3K4, mono‐ and di‐methylated forms of H3K27 are present (Le et al. 2025). H3K27me3 is essential for development and marks genes that are repressed but can be induced upon appropriate cues (Yamaguchi 2021). Polycomb Repressive Complex 2 (PRC2), a large protein complex, mediates the deposition of H3K27me3 (Bieluszewski et al. 2021; Le et al. 2025). However, in a broad range of eukaryotes, such as *Phaeodactylum tricornutum* , PRC2 represses TEs by depositing H3K27me3 (Hisanaga et al. 2023). Several stress types have been reported to alter H3K27me3 levels, for example, nitrogen supply triggers H3K27me3 enrichment at nitrogen‐responsive loci (Widiez et al. 2011), whereas UV‐B light disrupts flowering due to its loss from FLC, the repressor of flowering (Dotto et al. 2018). H3K27me3 demethylases (e.g., JMJ30) are usually induced upon ABA treatment or abiotic stresses and are necessary for heat acclimation (Yamaguchi et al. 2021; Nunez‐Vazquez et al. 2022). Yet, H3K27me3 depletion is not essential for the upregulation of cold‐induced genes (Faivre et al. 2024). Priming shade treatment results in removal of H3K27me3 from the shade‐memory‐related genes (Cheng et al. 2024). Although H3K9me2 is rather associated with TEs and repetitive loci (Mao et al. 2015), the occupation of drought‐responsive loci by an H3K9me1/2 demethylase induces drought response in *Arabidopsis* (Wang et al. 2021), suggesting the role of constitutive heterochromatin marks in stress response.

### 3.3 ChIP‐Analysis of Histone Modifications in Plants Under Stress

Chromatin architecture undergoes dramatic remodeling across the distinct phases of plant stress response. During stress, chromatin is relaxed and then becomes compact after recovery (Lamelas et al. 2020). Histones H2A and H2B are evicted upon extensive RNA polymerase activity (Shaytan et al. 2015) and accumulate in plants under heat stress (Lamelas et al. 2020). The comprehensive work by Lamelas et al. (2020) was performed using proteomics and transcriptomics, whereas several papers report the distribution of histones and their modifications in plants under stress using ChIP. For instance, Lang‐Mladek et al. (2010) demonstrated that the heat‐ or UV‐induced activation of a transgene locus occurred through H3K9ac. Sani et al. (2013) observed the loss of H3K27me3 from salt‐responsive genes in hyperosmotic primed *Arabidopsis thaliana* plants. Zhu et al. (2023) reported the accumulation and removal of H3K27me3 2 and 24 h after chilling, respectively. Temel et al. (2017) reported the accumulation of H3K9ac, H3K4me3, and H3 at the *hsp17* gene, which was induced two‐fold by drought. Bertini et al. (2018) showed that methyl jasmonate priming resulted in the accumulation of H3K9ac on defense‐related genes in wounded rice plants. Yung et al. (2022) reported that salinity priming caused alterations in histone marks H3K4me2, H3K4me3, and H3K9ac. Pratx et al. (2023) showed lower histone turnover, the retention of H3.3, a histone variant, and accumulation of H3K4me3 on heat stress memory genes.

## 4 ChIP

### 4.1 Methodology

The interaction of proteins with DNA at particular regions is vital for both chromatin structure and cellular functions such as replication and transcription. ChIP remains the most‐used method to study DNA‐protein interactions and histone modifications (Zhu et al. 2024). Commercial kits and methodological advancements shown by the accumulation of numerous publications have standardized ChIP as a routine method particularly in mammalian cells (Egelhofer et al. 2011; Wardle and Tan 2015). The technique involves several major steps such as fixation, chromatin extraction, sonication, immunoprecipitation (IP), and DNA purification. Analysis of the recovered DNA can be done by conventional or quantitative PCR, sequencing, or microarray. Beyond DNA‐protein interactions, RNA‐protein interactions can also be investigated by ChIP (Das et al. 2004).

The first yet optional step in ChIP experiments is the immobilization of protein‐DNA interactions. To ensure that these interactions are kept stable, biological material, for example, cell cultures or leaf samples, is cross‐linked to preserve protein–nucleic acid interactions. Usually, formaldehyde is preferred for cross‐linking as it can penetrate into tissues and binds proteins to nucleic acids, as well as to other proteins. Its cross‐linking effect can be quenched simply by the addition of a freshly prepared glycine solution to the medium and reversed by heating (Das et al. 2004). Typically, plant material is ground in liquid nitrogen, homogenized in an appropriate buffer, often enriched with Tris and/or sucrose, containing protease inhibitors and reducing agents (e.g., β‐mercaptoethanol), and then filtered (e.g., through a few layers of Miracloth). Following extractions using buffers containing sucrose and vigorous centrifugation steps, the cell pellet is finally dissolved in a detergent‐containing buffer for nucleus lysis (Temel et al. 2017).

Once the nucleus lysis is complete, chromatin must be sheared into DNA fragments ranging from 500 to 1000 bp. This is usually achieved via sonication. As an alternative, enzymatic digestion using micrococcal nuclease (MN) leaves nucleosomes intact and, therefore, creates consistent results, that is, 146 bp fragments (Das et al. 2004). Before the IP step, the chromatin sample is cleared by centrifugation and the resulting supernatant is diluted. For IP, the chromatin sample is incubated with the antibody specific to the protein of interest in a rotating wheel. IP mixture should also contain agarose or magnetic beads conjugated to the Protein A or Protein G, which have affinity for antibodies. The reference sample is the input, which is not antibody‐treated and corresponds to the non‐IP fraction of the chromatin. The negative control is the mock, which is usually incubated with an IgG antibody. Samples that are not given any antibody can also serve as mock. IP samples and mock, but not input, are then washed with several buffers (i.e., low‐ and high‐salt buffers) to retrieve chromatin. Cross‐linking is reversed by heating the chromatin, protein impurities are eliminated and then DNA is eluted. Purified DNA fragments correspond to the genomic regions that are bound by the protein of interest and can be analyzed by PCR or sequencing (Temel et al. 2017).

### 4.2 Troubleshooting

Although ChIP is a powerful and well‐established method for determining the target regions of DNA‐binding proteins, it has many challenges. As a general rule, all steps from extraction onward should be carried out under cold conditions to prevent warming of samples or buffers. One of the challenges is the optimization of cross‐linking. While native ChIP, which omits the cross‐linking step, might be suitable for histone studies, the IP of the proteins indirectly and/or transiently bound to DNA requires cross‐linking (Collas 2009; Small et al. 2021). Cross‐linking also stabilizes transient protein‐DNA interactions (Massie and Mills 2009). Moreover, cross‐linking of proteins residing in large complexes requires the use of a protein–protein cross‐linking agent (e.g., dimethyl adipimidate) prior to formaldehyde treatment as such proteins might be distant from DNA (Kurdistani and Grunstein 2003). Its duration should be optimized to prevent over‐cross‐linking, which can mask epitopes, impair sonication, DNA isolation, and de‐crosslinking, which corresponds to reversal of cross‐linking (Johnson and Bresnick 2002; Das et al. 2004; Haring et al. 2007; Zhao et al. 2020). Histones are prone to over‐cross‐linking as formaldehyde, particularly, targets lysine residues (Das et al. 2004). Penetration of formaldehyde into plant tissues requires the use of vacuum (Saleh et al. 2008).

As is common in any method using plant material, cell lysis and chromatin extraction are problematic steps in ChIP experiments due to the presence of numerous secondary metabolites, rigid cell walls, and large vacuoles. Grinding plant tissues, especially when processing multiple samples at the same time, is time‐consuming and requires maintaining a cool environment. Plant material should be crushed thoroughly into a fine powder and kept cool. The resulting ground material should be immediately mixed with the cool extraction buffer. All extraction buffers should be supplemented with protease inhibitors such as phenylmethanesulfonyl fluoride (Saleh et al. 2008). To ensure the stability of the inhibitors, protease inhibitor cocktail tablets can be utilized. Use of protease inhibitors in tablet form also reduces preparation time (Temel et al. 2017). Certain plant tissues, such as seed and fruit, are particularly problematic due to their cell wall structure and cellular constituents. However, optimized buffers containing sodium deoxycholate, IGEPAL CA‐630, and hexanediol, combined with adjusted centrifugation steps, can ensure efficient chromatin isolation and IP (Zhang et al. 2024).

The outcome of sonication is determined by several factors, such as sonication strength and duration, and sample temperature, and therefore requires optimization. Use of a focused ultrasonicator, such as Covaris, can generate reproducible results in shorter durations. An aliquot of the sonicated sample should be checked using gel electrophoresis or BioAnalyzer to prevent waste of material and antibody (Saleh et al. 2008). DNA smear obtained from the sonicated samples should be concentrated around 500 bp, indicating appropriate fragmentation (Temel et al. 2017).

The amount of chromatin in samples to be compared should be normalized and can be measured with a Bioanalyzer or Qubit (Cayir et al. 2024; Lucini et al. 2024). The interaction between the antibody and its target protein bound to chromatin occurs on either agarose or magnetic beads, while the latter, due to their uniform size, smooth surface and increased stability, offer an alternative to agarose beads, which are highly labile and not very user‐friendly (Schmidt et al. 2009; Zhu et al. 2024).

Antibody specificity is arguably the most crucial issue in ChIP experiments and its validation might be, particularly, challenging for histone modifications (Small et al. 2021). Polyclonal antibodies are usually preferred for ChIP, as they recognize multiple epitopes, unlike monoclonal antibodies, which target a single epitope. Polyclonal antibodies are heterogeneous and prone to lot‐to‐lot variation (Das et al. 2004; Wardle and Tan 2015). As with antibody‐based methods, not all antibodies yield the expected results. Although most commercial antibodies are now tested across various species and cell/tissue types, an antibody validated for efficiency in one species might not work in the same or a closely related species (Temel A, unpublished data). The optimal amounts of antibody and chromatin should be determined empirically. Antibodies developed to recognize modified histones or modified nonhistone proteins (e.g., phosphorylated RNA polymerase II) are commercially available (Temel et al. 2017; Chen et al. 2019). Cross‐reactivity of antibodies is particularly problematic when dealing with histone modifications (Egelhofer et al. 2011). The best way to achieve reproducibility is to indicate the lot and catalog number of the antibody used, as suggested by Wardle and Tan (2015). Furthermore, the use of barcodes allows for the analysis of multiple samples within a single IP. By processing barcoded chromatin samples simultaneously, technical variation between samples is minimized. However, a drawback of this strategy is the utilization of crude chromatin samples for barcoding of DNA fragments. The integrity of the chromatin might be compromised during this step (Kumar et al. 2025).

### 4.3 Alternatives of ChIP

ChIP has been utilized for decades and merely modified for its readout approaches, for example, PCR and sequencing (Solomon and Varshavsky 1985). Nevertheless, it has several alternatives, such as cleavage under targets and release using nuclease (CUT&RUN) and cleavage under targets and tagmentation (CUT&TAG), both of which were developed by Steven Henikoff's group (Ahmad and Henikoff 2025). CUT&RUN does not require crosslinking and marks the protein of interest using a primary antibody, which is recognized by protein A fused to MN. Instead of isolating and fragmenting chromatin, cells are incubated with special magnetic beads, immobilizing them before being recognized by an antibody. Membrane permeabilization is achieved by digitonin treatment. Duration times are shorter and background is lower than in ChIP. CUT&RUN is suitable for low‐cell‐number applications (Skene et al. 2018). Henikoff's group also made numerous improvements to the original method, such as facilitation of the fusion protein isolation (Meers et al. 2019). In order to accelerate the existing protocol, Kaya‐Okur et al. (2019) replaced MN with a hyperactive Tn5 transposase, which ligates the sequencing adaptors to the flanking regions of the protein‐binding site, and introduced this approach as CUT&TAG. They also advised using a secondary antibody to attract more protein A to the region of interest. Very recently, the same group developed Cleavage Under Targets & Targeted Integration via Methyltransferase Expression (CUT&TIME) to capture the history of the open chromatin. CUT&TIME relies on transient expression of a 6‐methyl adenosine (6 mA) methyltransferase (MTase), which introduces 6 mA to open chromatin, in progenitor cells. The progeny of the transfected cells develop with m6A‐containing genomes and exhibit no abnormal development after 12 days. Finally, differentiated cells are analyzed by CUT&TAG at the single‐cell level. With this method, open regions and the corresponding genes of a developmental program can be identified (Eldred et al. 2025).

The limitations of ChIP are further exacerbated in plants. However, the techniques that were briefly described above as alternatives to ChIP have not been widely employed in plants. One of the challenges is the isolation of high‐quality, intact nuclei from plant tissues, which have cell walls. The use of proper buffers can help isolate high‐quality nuclei with high yield (Ouyang et al. 2021; Fu et al. 2024). Yet, there are successful results. For instance, CUT&RUN was used to demonstrate the changes in H3K27me3 landscape in 6C endosperm nuclei obtained from mutant Arabidopsis seeds (Choi and Gehring 2024). Xu et al. (2026) applied CUT&RUN in tomato plants and reported the changes in genome localization of JmjC domain‐containing protein and MYC2 protein. An exhaustive characterization of these emerging techniques is beyond the scope of this review. Otherwise, there are excellent review and protocol papers dealing with newer chromatin‐profiling methods (Skene et al. 2018; Kaya‐Okur et al. 2019; Eldred et al. 2025; Yang et al. 2025).

## 5 Conclusions and Future Prospects

Stress‐responses include the accumulation of gene transcripts and metabolites and are mediated, or affected, by a vast array of epigenetic modifications. Plants' ability to retain changes and store information after the stress exposure is called plant stress memory. The information generated by the stress can confer resilience against an upcoming stress. Stress memory is mainly based on the imprints on the chromatin structure, including histone modifications (Liu, Able, et al. 2022). Priming is the pathway dictated by stress memory and includes the regulation of gene expression (Harris et al. 2023). The reprogramming of the transcriptional machinery requires the alteration of chromatin structure; yet, priming is often associated with chromatin changes independent of transcription (Bhadouriya et al. 2021), with histone being a prominent feature of chromatin structure (Bhadouriya et al. 2021). Understanding the stress responses and how these responses are kept and transmitted is crucial for the maintenance and the enhancement of plant yield, which is threatened by climate change. Identification of epigenetic changes, particularly simultaneous analysis of several histone modifications, can establish a signature of stress tolerance and might be useful for breeding purposes through selection of elite genotypes and identification or synthesis of more effective priming agents (Liu, Wang, et al. 2022; Aswathi et al. 2025). Although heavily studied, priming has not yet been thoroughly utilized in agriculture due to its highly complex nature (Aswathi et al. 2025).

Simultaneous analysis of several histone modifications can establish a signature of stress tolerance and more effective priming agents (Liu, Wang, et al. 2022). Better characterization of histone modifications in primed plants might pave the way for epigenome editing, which modifies the epigenetic status of a given gene (Qi et al. 2023; Zhang and Zhu 2025). Site‐specific manipulations that target changes in chromatin structure can better explain whether epigenetic modifications in particular loci have causative effects in priming (Oberkofler and Bäurle 2022; Harris et al. 2023). Development of faster and/or more sensitive ChIP protocols or commercial kits can enrich our understanding of histone modifications associated with stress response and/or tolerance. Single‐cell approaches might address epigenetic heterogeneity and result in more detailed and reliable outcomes. There are also numerous ChIP alternatives that await utilization in plant studies. Studies in plant systems are primarily focused on acetylation and methylation of histones; yet, there might be unexplored histone marks associated with stress response and/or tolerance in plants. Several histone marks and/or variants affect each other and act together; for this reason, characterization and interpretation of histone code along other epigenetic modifications such as DNA methylation will be of interest. In addition to ChIP, high‐throughput proteomics and machine learning (ML) methods are necessary to predict the phenotypic outcomes resulting from particular epigenetic compositions. Several ML approaches have been developed to predict gene expression and chromatin states from PTM data (Singh et al. 2016; Wen et al. 2024). Deep learning algorithms are implemented in mass spectrometry (MS)‐based proteomics to reduce processing time and optimize data acquisition (Wei et al. 2025). MS enables the high‐resolution identification of histone PTMs and, when coupled with ChIP, allows the definition of the proteins associated with particular histone PTMs, for example, H3K27ac (Scheid et al. 2022; Brun et al. 2026). The mechanisms by which external signals modulate the epigenetic landscape have not been fully understood.

## Author Contributions

A.T. and N.G.‐S. wrote the manuscript and reviewed it. N.G.‐S. did the figures.

## Conflicts of Interest

The authors declare no conflicts of interest.

## Data Availability

Data sharing is not applicable to this article as no new data were created or analyzed in this study.
